# Vaccination with a Human Papillomavirus L2 Multimer Provides Broad Protection against 17 Human Papillomavirus Types in the Mouse Cervicovaginal Challenge Model

**DOI:** 10.3390/vaccines12060689

**Published:** 2024-06-20

**Authors:** Zhenwei Han, Shen Wang, Ting Mu, Ping Zhao, Lingli Song, Ying Zhang, Jin Zhao, Wen Yin, Yue Wu, Huan Wang, Bo Gong, Min Ji, Richard B. S. Roden, Yanping Yang, Michel Klein, Ke Wu

**Affiliations:** 1 Project Management Department, Wuhan BravoVax Co., Ltd., Wuhan 430070, China; hanzhenwei@whu.edu.cn; 2 State Key Laboratory of Biocatalysis and Enzyme Engineering, Hubei Key Laboratory of Industrial Biotechnology, School of Life Sciences, Hubei University, Wuhan 430062, China; yinwen@hubu.edu.cn; 3 Regulatory and Medical Affairs Department, Wuhan BravoVax Co., Ltd., Wuhan 430070, China; shen.wang@bravovax.com (S.W.); lingli.song@bravovax.com (L.S.); gongbo325@gmail.com (B.G.); jimin@topgenebio.com (M.J.); 4 Innovative Discovery Department, Wuhan BravoVax Co., Ltd., Wuhan 430070, China; starbio520@gmail.com (T.M.); yingcensai1@hotmail.com (Y.Z.); wanghuanwill@hotmail.com (H.W.); 5 Test Development Department, Wuhan BravoVax Co., Ltd., Wuhan 430070, China; years6233@yahoo.com (P.Z.); yue.wu@bravovax.com (Y.W.); 6 Departments of Pathology, Oncology and Gynecology and Obstetrics, The Johns Hopkins University, Baltimore, MD 21287, USA; 7 Executive Office, Wuhan BravoVax Co., Ltd., Wuhan 430070, China; yan-ping.yang@bravobio.com; 8 Executive Office, Shanghai BravoBio Co., Ltd., Shanghai 200000, China

**Keywords:** human papillomavirus vaccine, L2, challenge study, *in vivo* infection model

## Abstract

Human papillomavirus (HPV) is a prevalent cause of mucosal and cutaneous infections and underlying conditions ranging from benign warts to anogenital and oropharyngeal cancers affecting both males and females, notably cervical cancer. Cervical cancer is the fourth leading cause of cancer deaths among women globally and is the most impactful in low- and middle-income countries (LMICs), where the costs of screening and licensed L1-based HPV vaccines pose significant barriers to comprehensive administration. Additionally, the licensed L1-based HPV vaccines fail to protect against all oncogenic HPV types. This study generated three independent lots of an L2-based target antigen (LBTA), which was engineered from conserved linear L2-protective epitopes (aa11–88) from five human alphapapillomavirus genotypes in *E. coli* under cGMP conditions and adjuvanted with aluminum phosphate. Vaccination of rabbits with LBTA generated high neutralizing antibody titers against all 17 HPV types tested, surpassing the nine types covered by Gardasil^®^9. Passive transfer of naïve mice with LBTA antiserum revealed its capacity to confer protection against vaginal challenge with all 17 αHPV types tested. LBTA shows stability at room temperature over >1 month. Standard *in vitro* and *in vivo* toxicology studies suggest a promising safety profile. These findings suggest LBTA’s promise as a next-generation vaccine with comprehensive coverage aimed at reducing the economic and healthcare burden of cervical and other HPV+ cancers in LMICs, and it has received regulatory approval for a first-in-human clinical study (NCT05672966).

## 1. Introduction

Human papillomaviruses (HPVs) are near ubiquitous small DNA tumor viruses that infect basal keratinocytes of cutaneous and mucosal epithelia [1]. There are approximately 220 types of HPVs that have been classified into five major genera: alpha, beta, gamma, mu, and nu [2]. Most HPV infections are asymptomatic and result in no clinical disease or produce benign skin, laryngeal, or anogenital warts, but a subset are oncogenic [3]. The about 40 mucosal αHPV types [4,5] are subdivided into two categories: the high-risk (HR) HPVs with oncogenic potential and the low-risk (LR) HPVs associated with genital warts. A group of 12 sexually transmitted, high-risk alphapapillomaviruses (Group 1), including α9 HPV (16, 31, 33, 35, 52, and 58), α7 HPV (18, 39, 45, and 59), α5 HPV51, and α6 HPV56, are associated with cervical cancer and, with increasing evidence, other various types of anogenital and oropharyngeal cancers in both men and women [6,7]. While HPV types 16 and 18 are detected in 50% and 20% of all cervical cancers, respectively, Arbyn and colleagues observed that 1 or more of the 20 common HPV types were significantly more prevalent among cervical cancer cases compared to women with normal cervical cytology [8]. With approximately 600,000 new cases and 340,000 deaths in 2020, cervical cancer is the fourth most common cancer of women worldwide and is particularly impactful in low- and middle-income countries because of the limited access to screening and the current vaccines [9]. It is clearly important to protect against all HPV types that are drivers of cervical cancer. In addition to the dozen HR-HPVs (Group 1), another group of ten HPVs (HPV26, 30, 34, 53, 66, 68, 69, 70, 73, 82) have been classified as probably or possibly oncogenic [10,11]. Low-risk α10 HPVs (6 and 11), which cause anogenital warts and condylomata acuminatum, are also involved in recurrent respiratory papillomatosis [2,12] and can rarely exhibit malignant progression, e.g., Buschke–Lowenstein tumor and lung cancer [13].

The human betapapillomaviruses (βHPVs) typically produce only subclinical infections or flat warts of the skin. However, a subset of βHPVs contributes to the development of cutaneous squamous-cell carcinoma (CSCC) in areas exposed to UV irradiation in immunocompromised patients (e.g., organ transplant recipients, AIDS patients) or those with the rare genodermatosis epidermodysplasia verruciformis (EV) [14]. HPV5 and HPV8 are the most prevalent βHPV types, which are also associated with 90% of CSCCs of EV patients [15].

The *L1* gene of the 8 kb HPV genome encodes the major capsid protein L1, which self-assembles into empty virus-like particles (VLP) when overexpressed. The *L2* gene encodes the minor capsid protein, L2, which is critical for the infectious process. When co-expressed with L1, it is incorporated into the capsid, but L2 alone does not form VLP. Biochemical analyses and cryo-electron microscopy with image reconstruction have revealed that papillomavirus particles contain 360 L1 molecules and up to 72 copies of L2 [16,17]. Vaccination with L1 VLP induces high-titer neutralizing antibodies in serum and provides protection from infection by the genotype used to make the vaccine but generally not against other genotypes, i.e., type-restricted protection. Passive transfer of neutralizing antibodies into naïve hosts is sufficient to confer protection, suggesting they are responsible for the protection provided by active vaccination.

Because of the type-restricted nature of protection, current HPV vaccines are based on a mixture of the L1 VLPs of the most medically important HPV genotypes formulated with an alum-based adjuvant [16]. Six such L1-VLP-based HPV vaccines have been licensed. They include the three bivalent vaccines (Cervarix^®^ (Glaxo Smith Kline Biologicals, Rixensart, Belgium), Cecolin^®^ (Xiamen Innovax Biotech Co., Ltd., Xiamen, China), and Valrinvax^TM^ (Yuxi Zerun Biotech Co., Ltd., Yuxi, China)) composed of high-risk HPV16 and HPV18 L1 VLPs, along with the two quadrivalent vaccines (Gardasil^®^ (Merck & Co., Inc., Rahway, NJ, USA) and Cervavac (Serum Institute of India, Pune, India)) that also contain the two low-risk HPV6 and HPV11 L1-VLPs and the nonavalent Gardasil^®^9 vaccine, which incorporates additional L1 VLPs from HPV 31, 33, 45, 52, and 58 [18]. Except for Cecolin^®^ made in *E. coli*, the other vaccines are produced in yeast or baculovirus-infected insect cells (Cervarix^®^) [19,20]. TheGardasil^®^9 is safe and highly efficacious, providing protection against nearly 90% of cervical cancers; those targeting HPV6 and HPV11 protect against 90% of genital warts, too [21]. However, although Cervarix^®^ induces some cross-neutralizing antibody responses (albeit only against genotypes very closely related to the vaccine types like HPV31 and 45 [22]), the licensed vaccines protect against only a subset of cancer-causing HPV genotypes, and consequently, vaccinated women still need cervical screening. This is problematic because of the additional cost burdens on society and because the predictive power of cervical screening is greatly reduced. The inability to provide comprehensive coverage is also important because many patients are exposed to a diversity of HPV genotypes, in particular for women living with HIV who have high rates of multitype infections, including unusual types [23]. The complexity of manufacturing highly multivalent L1-VLP formulations also drives up costs, which may limit introduction in LMICs [24,25]. Finally, the licensed L1-VLP vaccines do not cover betapapillomaviruses that have been linked to cutaneous squamous-cell cancer (cSCC) and are particularly problematic in organ transplant recipients, HIV+, and other immunocompromised patients. These limitations justify the need for the development of a new generation of simplified, low-cost, and more broadly protective vaccines.

Vaccination with L2 induces neutralizing antibodies, although substantially lower titer than those produced by L1 VLP vaccines [16,26]. However, in contrast to L1 VLP, L2-based vaccine candidates can induce cross-type neutralizing and broadly protective immunity in animal models. This reflects the presence in the N-terminal region of L2 of several overlapping, linear, highly conserved neutralization epitopes (aa17–36, 20–38, 28–42, 56–75, 64–81, 96–115, and 108–120) [27,28], and this suggests the potential for developing a comprehensive HPV vaccine using this immunogen.

Following L1-mediated HPV binding to the heparan sulfate proteoglycans of the epithelial basal membrane, a conformational change of the capsid exposes L2’s positively charged N-terminal amino acids. This permits their cleavage by extracellular furin and results in the transient accessibility of L2’s conserved epitopes to neutralizing antibodies [29].

Several strategies have been designed to produce L2-based HPV vaccines, many of which utilize repetitive display of its conserved epitopes to boost their immunogenicity. They include the insertion of L2 neutralization epitopes into immunodominant surface loops of HPV L1 VLP and other viral or bacteriophages capsids, or their linkage with TLR ligands or a thioredoxin scaffold, and their fusion as L2 concatemers [26,27,28]. In this regard, the simple fusion of a limited number of L2 neutralization epitopes to produce single-chain multimers in *E. coli* has promise as a low-cost, broad-spectrum HPV vaccine [30]. Jagu et al. [31] generated such a recombinant multimer in *E. coli* by fusing the N-terminal region (aa11–88) from 5 HPV genotypes (6, 16, 18, 31, 39), termed “a11–88x5,” and thus presented four major neutralization epitopes (aa17–36, 28–42, 56–75, 64–81) of each. Vaccination with the L2 a11–88x5 protein formulated on aluminum phosphate adjuvant elicited serum antibodies that cross-neutralized all eleven homologous and heterologous αHPVs (6, 16, 26, 31, 33, 35, 45, 51, 56, 58, 59) tested and protected mice against vaginal challenge with pseudovirions for each of these genotypes [32,33].

The objective of this study was to fully characterize the α11–88x5 multimeric HPV vaccine (LBTA), optimize its production in *E. coli* under cGMP conditions, assess its thermostability and safety, and evaluate its protective efficacy against an expanded spectrum of 17 divergent HPV genotypes. The LTBA drug product is essentially the same protein as a11–88x5 but is given a different name to recognize a distinct production procedure as compared to the earlier product, which may influence its stability and immunogenicity. Our findings suggest that the LBTA adjuvanted in aluminum phosphate has potential as a next-generation affordable vaccine against a broad spectrum of mucosal and cutaneous HPV infections.

## 2. Materials and Methods

### 2.1. LBTA Multimer [a11–88x5] Production, Purification, Characterization, and Clinical Lot Preparation

L2 N-terminal residues 11 to 88 derived from HPV6, HPV16, HPV18, HPV31, and HPV39 were linked to engineer a fusion peptide named L2-based target antigen (LBTA) [32]. The *LBTA* gene sequence was codon-optimized for *E. coli* expression and inserted into the pET28a vector (Novagen, San Diego, CA, USA). The vector was then transformed into Rosetta^TM^ 2(DE3) pLysS Competent Cells (EMD Millipore Corporation, Burlington, MA, USA) and induced with 1mM IPTG. After harvesting, bacteria were lysed using a high-pressure homogenizer, and inclusion bodies were collected, washed, solubilized in 8 M urea, and subjected to a two-step chromatography process in the presence of urea. Subsequently, on-column refolding followed by dialysis was performed to obtain the LBTA drug substance (DS). The protein concentration of LBTA DS was adjusted based on the desired dose of final drug product (120 µg or 240 µg per dose). Sucrose was added to a final concentration of 20% (*w*/*v*) as a stabilizer. The material was sterilized through a 0.22 µm filter, filled into glass vials, and then lyophilized to obtain an LBTA intermediate drug product. Finally, the lyophilized LBTA was reconstituted in 20 mM sodium bicarbonate buffer (pH 8.0 ± 0.5) containing 100 mM of sodium chloride and adsorbed onto 500 µg of aluminum phosphate (AlPO_4_) to obtain the final LBTA drug product (LBTA DP).

The LBTA DS was thoroughly characterized before and after lyophilization. The protein concentration was measured using the Lowry method. The endotoxin content in the vaccine sample was measured by the Limulus Amebocyte Lysate (LAL) assay. LBTA purity was assessed using SDS-PAGE and RP-HPLC analysis. Its mass was determined by extractive electrospray ionization time-of-flight mass spectrometry (ESI-TOF-MS), and its sequence was confirmed by N-terminal sequencing (Edman degradation), peptide mapping, and LC-ESI-MS/MS (Shanghai Applied Protein Technology Co., Ltd., Shanghai, China). The circular dichroism far-UV analysis of the L2 multimer was performed on a Chirascan v100 spectrometer at a concentration of 1.2 mg/mL. CDNN software 2.1 was used for deconvolution data analysis.

### 2.2. Stability Studies on LBTA cGMP Clinical Lots

The LBTA DS (before lyophilization) was stored at ≤−60 °C, while the LBTA intermediate drug product (i.e., the lyophilized powder) was stored at 5 ± 3 °C. Real-time stability studies were carried out. The analytical characterization for LBTA DS included protein and antigen content, % of protein purity (as an indication of potential degradation), pH, and residual endotoxin at each given time point. The analytical characterization of LBTA intermediate drug product included protein content, >90% of protein purity, residual moisture, osmolarity, pH, and >80% of adsorption to AlPO_4_.

Since the final LBTA vaccine is intended to be a mix-and-inject format, in-use stability studies were performed to ensure that the products would be stable after reconstitution at the clinical sites (i.e., reconstitution of LBTA lyophilized powder with a buffer containing 500 µg aluminum phosphate gel). For in-use stability, three cGMP lots of lyophilized LBTA were mixed with reconstitution buffer and tested at the specified storage temperatures and conditions (5 ± 3 °C up to 4 h; 25 ± 2 °C up to 2 h; or 37 ± 2 °C up to 2 h) for their protein content and % of AlPO_4_ adsorption.

### 2.3. Toxicology Studies of LBTA cGMP Clinical Lots

Three *in vivo* GLP toxicology studies were performed by Medicilon Preclinical Research, LLC (Shanghai, China). They included a single-dose acute toxicity study and a repeat-dose toxicity study in Sprague Dawley (SD) rats, as well as an active systemic anaphylaxis test in guinea pigs. In addition, an *in vitro* hemolysis test was performed under GLP conditions using rabbit red blood cells.

### 2.4. Determination of Percent LBTA Adsorption onto Aluminum Phosphate

The lyophilized LBTA was mixed with AlPO_4_ or water, respectively. The sample mixed with AlPO_4_ was centrifuged for 2 min at 230× *g*, and its supernatant was analyzed for unbound protein content by the Lowry assay [34]. The lyophilized sample dissolved in water was used to determine total protein content. Percent (%) adsorption was calculated as [(total protein − unbound)/total protein] × 100.

### 2.5. Immunization of Rabbits

**Figure 1:**
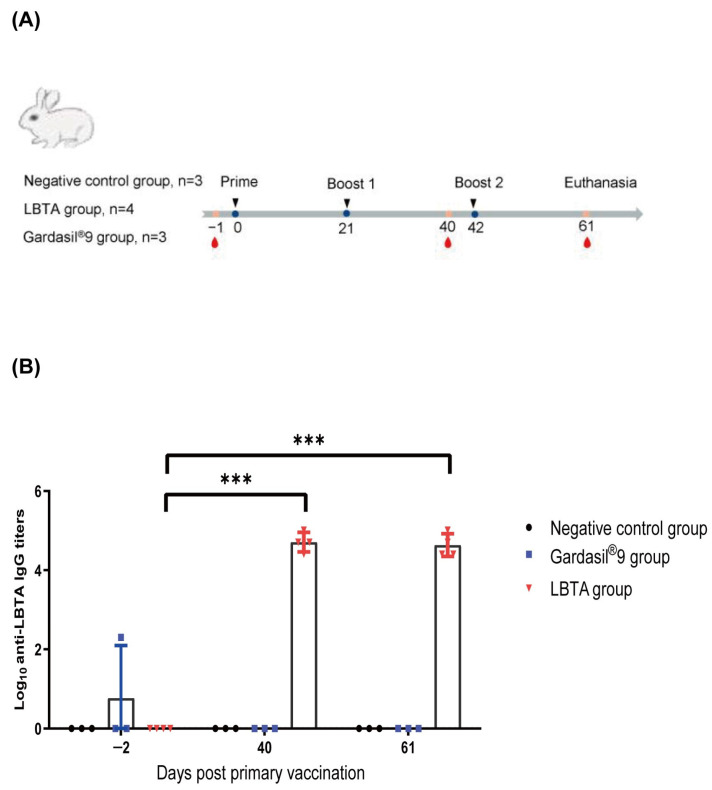
LBTA vaccination induced robust antibody titers against LBTA in rabbits. (**A**) Schedule of vaccine administration and blood collection. Dosing (black triangles) and terminal blood collections (red drop shapes) were carried out at the indicated times. (**B**) Binding IgG antibody titers against LBTA were determined by indirect ELISA for sera collected on D-2, D40, and D61 after primary vaccination. *** *p* < 0.001.

The pre-clinical animal studies were performed using research-grade LBTA produced using the same process as that of the cGMP clinical materials. Ten clean-grade female New Zealand white rabbits weighing 2.0–2.5 kg were purchased from Wuhan Yizhicheng Biotech Co., Ltd., Wuhan, China. The animals were housed, and all procedures were conducted at Wuhan Hualian Biotechnology Co. in Wuhan, China, with prior approval from the animal ethics committee (No. HLK-20200605-001). After a quarantine period, the rabbits were randomly divided into three groups: negative control group (n = 3), LBTA group (n = 4), and Gardasil^®^9 group (n = 3). The negative control group received an injection of 0.9% NaCl. The LBTA group rabbits were vaccinated with 125 µg LBTA and 500 µg aluminum phosphate adjuvant (AlPO_4_), while the Gardasil^®^9 group rabbits received the human dose of the licensed vaccine. All animals were vaccinated using a three-dose regimen on day 0 (D0), day 21 (D21), and day 42 (D42). Blood samples were collected 2 days before priming (D-2) and 40 days (D40) or 61 days (D61) after the first injection. Serum-binding antibody titers were measured at each time point, and serum-neutralizing antibody titers were measured on D61 (Figure 1A).

### 2.6. Qualitative Antigenicity Assay

A sandwich ELISA was used to assess the antigenicity of the LBTA drug substance (DS) based on the presence of two different neutralization epitopes (HPV L2 aa17–36 and aa58–64) in the L2 multimer. These epitopes are recognized by the chimeric monoclonal antibodies JWW1 and JWW2, respectively [35]. Additionally, 96-well plates were coated with 0.5 mg/well of JWW2 capture monoclonal antibody at 2–8 °C overnight. Plates were blocked with 10% skimmed milk and washed with PBS-Tween20 (PBS-T). The test samples were added to the plates, then incubated at 37 °C for 1 h, and plates were washed three times with PBS with Triton X-100 (PBS-T) (Thermo Fisher Scientific, Waltham, MA, USA). The HRP-conjugated JWW1 antibody was then added to the plates. Following a 1 h incubation at 37 °C and three PBS-T washes, TMB (3,3′,5,5′ tetramethylbenzidine) was dispensed in the wells. The plates were then incubated at room temperature for 15 min, the reaction stopped with 1M sulfuric acid, and the absorbance was measured at 450 nm. A positive colorimetric reaction indicated that both neutralization epitopes were surface exposed.

### 2.7. LBTA-Binding Antibody Titration by Enzyme-Linked Immunosorbent Assays

Binding antibody titers against the LBTA immunogen were determined by ELISA. Additionally, 96-well plates were coated overnight with 0.2 µg/well of purified LBTA antigen in bicarbonate buffer (pH 9.6) at 2–8 °C. After washing the plates with PBS-T, wells were blocked with 10% skimmed milk for 2 h at 37 °C. Starting at a 1:100 dilution, rabbit serum samples were 4-fold serially diluted with blocking buffer and added to the plates. Blank, negative, and positive controls were included in parallel. After incubation for one hour at 37 °C, the plates were washed 3 times with PBS-T. Then, 100 mL of HRP-conjugated goat anti-rabbit antibody were added to the wells, and plates were further incubated for another hour at 37 °C. TMB (100 mL/well) was then added for color development, and the reactions were stopped with 100 mL of 1M sulfuric acid. Absorbance values were read at 450 nm, and the cutoff value was determined. The cutoff value was calculated as being 2.1 times the mean OD_450_ values obtained for samples from the negative control. The endpoint titer of serum samples was determined as the maximum dilution fold when the OD_450_ value was equal to or greater than the cutoff value.

### 2.8. HPV Pseudovirion (PsV) Preparation

The 293TT cells (ATCC, CRL-3467, overexpressing SV40 T antigen and obtained from John Schiller, NCI, NIH, Bethesda, MD, USA) (3.5 × 10^6^/20 mL) were cultured in T75 cell culture flasks in DMEM-10 medium (DMEM with 10% fetal calf serum, 1% non-essential amino acids, 1% Glutamax-I) at 37 °C and 5% CO_2_ for 16–18 h until they reached 40%~60% confluence. Then, 19 μg of a firefly luciferase reporter gene plasmid and 19 μg of an HPV serotype-specific L1/L2 co-expression plasmid were transfected into 293TT cells using 76 mg polyethyleneimine (PEI) (Linear, MW 25,000). The cells were cultured at 37 °C in 5% CO2 before the medium was replaced 6 h after transfection. After 72 h, the transfected cells were submitted to enzymatic digestion with 0.05% trypsin-EDTA (Gibco, Billings, MT, USA, 25300-062) and harvested by centrifugation at 200× *g* for 5 min at 4 °C. The cell pellet was washed twice with ice-cold Dulbecco’s PBS (DPBS) (Gibco, 14040-133) and then resuspended with 1.4 times the pellet volume of DPBS supplemented with 9.5 mM MgCl_2_ to promote the maturation of the pseudotyped virus (PsV).

To prepare the cell lysate, 10% Triton X-100 (Thermo, 85111), Benzonase (Sigma, St. Louis, MO, USA, E1014-25KU), Plasmid-Safe ATP-dependent DNase (Lucigen, Middleton, MA, USA, E0054-10D1), and 1 M (NH4)_2_SO_4_ (pH 9.0) were added to the cell suspension to a final concentration of 0.5% Triton X-100, 0.1% Benzonase, 0.1% Plasmid-Safe ATP-dependent DNase, and 25 μM of (NH4)_2_SO_4,_ respectively. The cell lysate was then incubated for 24 h at 37 °C. After incubation, it was cooled on ice for 5 min, and then a volume of 5 M NaCl equal to 0.17 times that of the cell lysate was added to a final concentration of 0.850 M NaCl. The mixture was centrifuged at 5000× *g* for 5 min at 2 °C to 8 °C. The resulting supernatant (Supernatant #1) was collected, and the remaining pellet was resuspended in a volume of DPBS/0.8M NaCl equal to that of the cell lysate. The resuspended pellet mixture was centrifuged again at 5000× *g* at 2 °C to 8 °C for 5 min, and the resulting supernatant (Supernatant #2) was collected and combined with Supernatant #1. The mixture (i.e., Supernatant #1 plus Supernatant #2) was centrifuged again at 5000× *g* at 2 °C to 8 °C for 5 min. The resulting supernatant containing the PsVs was collected, and pseudovirions were analyzed by transmission electron microscopy, titrated, and stored at ≤60 °C.

The 293TT cells, the luciferase reporter gene plasmid, and the L1/L2 expression plasmids were kindly provided by the National Institutes of Health (USA) and Dr. R.B. Roden (Johns Hopkins University).

### 2.9. Transmission Electron Microscopy (TEM) Imaging of HPV Pseudovirions

Image analysis of pseudovirions was performed by Servicebio (Wuhan, China). Briefly, 20 µL of pseudovirion preparation in Dulbecco’s phosphate-buffered saline (0.8 M sodium chloride) was placed onto a carbon-coated 150 mesh copper grid. Excess liquid was removed using filter paper. Phosphotungstic acid (2%) was added as negative stain for 1–2 min. The grid was dried at room temperature and then examined under 80 kV accelerating voltage in a transmission electron microscope (TEM) HT7800/HT7700 (HITACHI, Tokyo, Japan).

### 2.10. HPV Pseudovirion (PsV) Titration

The 293TT cells (1 × 10^5^ cells/well) were dispensed in 0.5 mL of DMEM-10 in a 96-well plate (Corning, Corning, NY, USA, Cat# 3599) and incubated overnight at 37 °C and 5% CO_2_. The PsV samples at a starting dilution of 1:100 were 10-fold serially diluted with DMEM (10%FBS, 1% MEM NEAA (Gibco, Cat#10370088), 1% GlutaMAX™ (Gibco, Cat#41090036)). Then, 100 µL of diluted PsV samples were added to the 96-well plates. DMEM (10%FBS, 1% MEM NEAA, 1% GlutaMAX™) was used as negative control. The plates were incubated for 44 to 52 h at 37 °C and 5% CO_2_. The cells were lysed, and the luciferase activity was measured by the BriteLite Plus Reporter Gene Assay System (PerkinElmer, Waltham, MA, USA). The relative light unit (RLU) values were read using a chemiluminescence detector (EnSight, PerkinElmer). Each PsV sample was assayed at ten dilutions in eight duplicates. The cutoff value was calculated as being 10 times the mean RLU values obtained for samples from the negative control. The median tissue culture infectious dose (TCID_50_) was calculated using the Spearman–Karber method.

### 2.11. In Vitro HPV Pseudovirion-Based Neutralization Assay (PBNA)

The ability of rabbit antisera to neutralize 17 HPV types was assessed in an *in vitro* HPV pseudovirion-based neutralization assay [36] with some modifications. CHO-furin cells (from J. Schiller, NCI, NIH) (1 × 10^6^ cells/17mL) were plated in a T75 cell culture flask with DMEM (10% FBS, 0.2 mM/L L-Proline, 1xPenStrep) and incubated for 96 ± 4 h at 37 °C in 5% CO_2_. The cell supernatant was collected by centrifugation at 350× *g* for 5 min. MCF10A cells (ATCC, CRL-10317) (2 × 10^4^ cells/well) in MEGM (Mammary Epithelial Cell Growth Medium) (Lonza, Basel, Switzerland, Cat#CC-3150) were dispensed in 96-well plates (Corning, Cat# 3599) and cultured at 37 °C, 5% CO_2_, for 24 ± 2 h until a dense monolayer was formed. After three washes with PBS, 50 mL of cell lysis buffer (PBS containing 0.5% Triton X and 0.1 M ammonia) was added, and plates were incubated at room temperature for 5–7 min until cells were completely lysed. Plates were then washed with PBS several times to prepare extracellular matrix (ECM)-coated plates. Then, 50 mL of HPV PsV (2 × 10^3^ to 2 × 10^4^ TCID_50_/mL) and 70 mL of CHO-furin cell culture supernatant containing 8.57 mg/mL heparin solution were added to ECM-coated plates and incubated overnight. Rabbit serum samples were pre-treated at 56 °C for 30 min, then 4-fold serially diluted with Ham’s F-12K (Kaighn’s) Medium (Gibco, Cat# 21127022) with 10% fetal bovine serum, 0.2 mmol/L L-Proline, and 1% penicillin–streptomycin–glutamine (100×) (Thermo Fisher) starting at a 1:50-fold dilution. Diluted serum samples (100 mL) were added to the plates and incubated at 37 °C in 5% CO_2_. Cell and virus controls were assayed in parallel. Four to six hours later, 50 mL/well of PGSA-745 adherent cells (ATCC, CRL-2242) (1.6 × 10^5^/mL) were added to the plates and incubated for 40–48 h at 37 °C in 5% CO_2_. After incubation, culture supernatants were discarded, and cells were lysed and analyzed for luciferase activity using a luciferase detection kit (PerkinElmer, 6066761). The relative light unit (RLU) values were read using a chemiluminescence detector (EnSight, PerkinElmer). The Reed–Muench method was used to calculate the 50% inhibitory concentration (IC_50_) value.

### 2.12. Establishment of the In Vivo Mouse Cervicovaginal Model of HPV Infection

The *in vivo* infection model for HPV was used to determine if antibodies produced in rabbits vaccinated with LBTA could provide passive protection to recipient mice, according to a published protocol [37] with minor modifications. The female BALB/c mice, aged six to eight weeks, received a subcutaneous injection of 3 mg of medroxyprogesterone acetate (Aladdin, Shanghai, China, M129409) in 0.5% carboxymethylcellulose (CMC, Sigma) four days before infection. After four days, vaginal smears were examined under a microscope (Leica Microsystems Co., Ltd., Wetzlar, Germany, DM1000) to confirm whether mice were in the diestrus phase. The vaginal lavage procedure involved using a 100 μL pipette to aspirate 30 μL phosphate-buffered saline (PBS) into the mouse vagina three times. After three vaginal instillation/aspiration cycles of PBS for each mouse, the fluid from the third aspiration was deposited onto a glass slide and fixed by immersing the air-dried vaginal smear slide in methanol for 3 min. After drying, the slide was stained with Wright-Giemsa Staining Solution A (Baso, Zhuhai, China, BA4017) for 15 s, followed by the addition of Wright-Giemsa Staining Solution B (Baso, BA4017). The slide was rinsed with water, dried naturally, and sealed with neutral mounting medium. Finally, the slide was scanned using a Leica microscope to assess the current estrus cycle stage based on cell imaging.

After entering the diestrus stage, mice were exposed to two cervical mechanical traumas with a Thinprep Cytologic Test (TCT) brush to create endometrial injury at four-hour intervals. Briefly, the TCT brush was inserted into the mouse vagina and rotated counterclockwise for 15 turns and clockwise for 15 turns to cause cervical mechanical trauma. The total pseudovirion inoculum volume was 25 μL per dose, composed of 20 μL of purified HPV pseudovirus carrying the luciferase reporter gene mixed with 5 μL of 3% carboxymethylcellulose-Na (CMC-Na, Sigma). The virus was delivered twice as a 12.5 μL inoculum dose containing 0.75 × 10^7^ TCID_50_ of pseudovirus. The mice were then infected intravaginally with 12.5 μL of HPV16 inoculum before undergoing the second round of mechanical trauma. Following a second trauma, the mice were re-infected with 12.5 μL of HPV16 inoculum intravaginally. Subsequently, the mice were placed in a handstand position for 5 min to ensure full delivery of the infecting pseudovirus. On Day 6 (48 h post-infection), the animals were anesthetized with isoflurane, and 20 μL of 30 mg/mL D-luciferin was administered intravaginally with a pipette. Two minutes later, the bioluminescence signal from the mouse cervix was monitored using an *in vivo* imaging system (IVIS) for small animals (IVIS Lumina Series III, PerkinElmer. The Living Image software 4.5.5 (Xenogen, PerkinElmer) was used to capture the region of interest (ROI) encompassing the luminescence emitting area of each mouse and calculate the average radiance in photons per second per square centimeter per steradian (p/s/cm^2^/sr).

This experiment was repeated 3 times to evaluate the reproducibility of the *in vivo* infection model. After showing its reproducibility and reliability, the model was used in HPV pseudovirus challenge experiments with 17 HPV types.

### 2.13. In Vivo HPV Pseudovirion Cervicovaginal Challenge in Mice

The HPV *in vivo* infection model and challenge experiments were approved by the animal ethics committee of Wuhan Hualian Biotechnology Co. (Wuhan, China) (No. HLK-20210301-001). Twenty-four female BALB/c mice were pre-treated with medroxyprogesterone acetate on Day 0 and divided into 4 groups (n = 6) for *in vivo* challenge with 17 HPV types. The groups included the LBTA, Gardasil^®^9, Saline, and control groups for each one of the 17 HPV types (HPV 6, 11, 16, 18, 31, 33, 45, 52, 58, 51, 56, 59, 35, 39, 66, 68, 73). On Day 3, mice in the LBTA, Gardasil^®^9, and Saline groups were passively immunized by intraperitoneal administration of 100 μL of sera from rabbits that had been vaccinated with either LBTA, Gardasil^®^9, or saline. The control group received no treatment. On Day 4, vaginal injury was conducted as described above, followed by vaginal challenge with each HPV pseudovirion type. Two days after the challenge, the mice were anesthetized using isoflurane gas anesthesia, and 20 μL of 30 mg/mL D-luciferin was instilled into the vagina with a pipette. Bioluminescence from the mouse cervix was measured after 2 min using IVIS. Pseudovirus vaginal challenges were performed with 17 HPV types, each mouse from the 17 individual experimental groups being infected with a single PsV dose of 10^6^–10^7^ TCID_50_. The challenge doses used per animal for each PsV serotype are listed in Supplementary Table S1.

### 2.14. Statistics and Data Analysis

The data were analyzed using the SPSS 23.0 software, and the results are presented as means ± standard deviation (SD). Statistical differences were determined using one-way ANOVA, with a significance level set at *p* < 0.05.

## 3. Results

### 3.1. L2 Multimer LBTA (a11–88x5) Production, Purification, Characterization, and Clinical Lot Preparation

**Figure 2:**
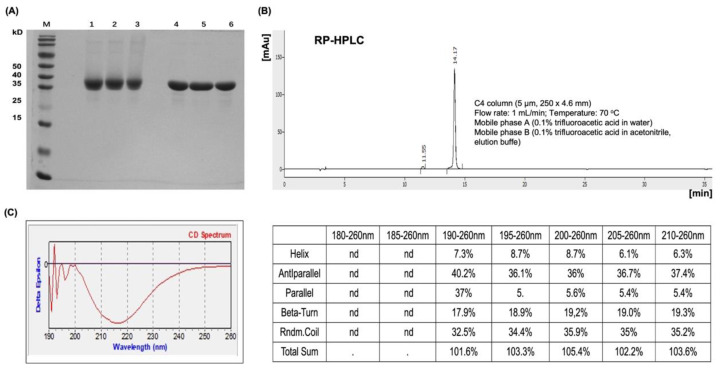
Characterization of LBTA. (**A**) SDS-PAGE analysis of three cGMP lots of purified LBTA using 8% polyacrylamide gels under non-reducing (lanes 1–3) and reducing (lanes 4–6) conditions. (**B**) Reversed-phase HPLC (RP-HPLC) analysis of protein purity by loading 40 μL of samples onto a C4 column (YMC-Triart Bio), with detection at a wavelength of 280 nm using a Photodiode-Array Detection (PDA) Detector. (**C**) Far-UV CD spectrum of LBTA. Deconvolution analysis of the main secondary structures, including α-helical and β-pleated sheet (including parallel and antiparallel structures) contents, as well as beta-turn and random coil components. nd, no data.

The gene coding for the linked N-terminal 11–88 amino acid residues from five different HPV (6,16,18,31,39) L2 proteins was cloned into pET28a to express the multiepitopic fusion protein LBTA (a11–88x5). After a two-step column-based purification from inclusion bodies and re-folding, the protein was shown to be ≥90% pure by SDS-PAGE and RP-HPLC analysis (Figure 2A,B), respectively. After cGMP production and purification, the LBTA yield obtained at the pilot scale from a 5L bioreactor was ≥2g/L of cell culture. Its molecular weight of ~40 kDa assessed by SDS-PAGE was confirmed to be 39.6 kDa by ESI-TOF mass spectrometry, and this is consistent with its theoretical molecular mass. The LBTA sequence was confirmed by N-terminal sequencing, peptide mapping, and LC-ESI-MS/MS after enzymatic digestion. The L2 neutralization epitopes aa17–36 and aa58–64 were exposed and recognized by their cognate-neutralizing monoclonal antibodies JWW1 and JWW2, respectively, in the qualitative antigenicity assay. The circular dichroism far-UV spectrum showed that the multimer contained 40% of the beta-pleated sheet structure, which was used as a marker of lot-to-lot consistency. The LPS content in the purified LBTA vaccine was less than 10 EU per human dose (at 120 µg or 240 µg). Residual bacterial DNA (<10 ng/dose) and protein contents (<0.1% of total protein) were below limits set by regulatory authorities.

### 3.2. Analysis of HPV Pseudovirion (PsV) Produced in HEK293TT Cells by Transmission Electron Microscopy (TEM)

Supplementary Figure S3 shows that PsV particles produced in HEK293TT cells have the well-preserved round shape structure of the virus with an average size of ~60 nm, displaying thus the typical morphodiagnostic features of HPV viruses [38] and HPV PsVs produced in HEK293TT cells by other investigators [39].

### 3.3. Stability and Toxicology Studies of LBTA cGMP Clinical Lots

The stability studies have shown that after 6 months, both the LBTA DS stored at ≤ −60 °C (Supplementary Table S2) and the lyophilized powder stored at 5 ± 3 °C (Supplementary Table S3) were stable as judged by protein content and purity. In addition, LBTA was recognized by the two selected neutralizing monoclonal antibodies (JWW1 and JWW2, respectively) following storage (i.e., DS at ≤−60 °C and DP at 5 ± 3 °C) at the latest test point (6 months). Furthermore, the vaccine was also found to be stable at room temperature and 37 °C when tested on day 28.

For in-use stability, three cGMP lots of LBTA drug products were mixed with the reconstitution buffer containing 500 µg aluminum phosphate and tested at the specified storage temperatures and conditions of 5 ± 3 °C up to 4 h, 25 ± 2 °C up to 2 h, or 37 ± 2 °C up to 2 h. Good stability was observed based on protein content and % of AlPO_4_ adsorption for all three cGMP lots tested (Supplementary Table S4). In addition, no visible particles were observed in the LBTA vaccine after reconstitution.

Toxicology studies, including single-dose toxicity, repeated-dose toxicity, active anaphylaxis, and *in vitro* hemolysis, showed that the LBTA vaccine had a good safety profile.

### 3.4. LBTA Induced Robust Binding and Cross-Neutralizing Antibody Responses in Rabbits against 17 Alphapapillomavirus Serotypes

We first compared LBTA immunogenicity with that of the licensed nonavalent vaccine, Gardasil^®^9, which targets HPVs 6, 11, 16, 18, 31, 33, 45, 52, and 58. Immunization of rabbits with 125 μg LBTA adjuvanted with 500 μg AlPO_4_ elicited very high levels of anti-LBTA-binding IgG antibody responses on D40 (19 days after the second administration) and D61 (19 days after the third administration) with GMTs of 51,200 and 43,054, respectively, while anti-LBTA-binding IgG antibody responses were virtually undetectable in the Gardasil^®^9 group and negative in the control group (Figure 1B). The lack of anti-LBTA antibody response to Gardasil^®^9 immunization was expected since Gardasil^®^9 is an L1 VLP-based vaccine that does not contain any L2 sequence. Anti-LBTA-binding IgG antibody levels in the LBTA group dramatically increased on D40 and D61 (*p* < 0.001, *p* < 0.001) compared with D-2. However, titers leveled off after the third administration (*p* > 0.05).

**Figure 3:**
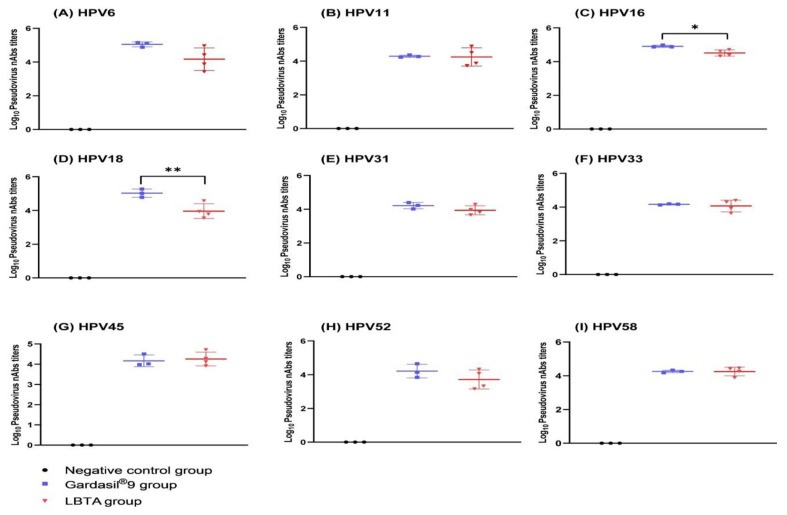
Cross-neutralizing antibody responses induced in rabbits on D61 after primary vaccination with Gardasil^®^9 and LBTA against the 9 HPV types included in Gardasil^®^9. (**A**) Pseudovirus nAbs against HPV type 6. (**B**) Pseudovirus nAbs against HPV type 11. (**C**) Pseudovirus nAbs against HPV type 16. (**D**) Pseudovirus nAbs against αHPV type 18. (**E**) Pseudovirus nAbs against HPV type 31. (**F**) Pseudovirus nAbs against αHPV type 33. (**G**) Pseudovirus nAbs against HPV type 45. (**H**) Pseudovirus nAbs against αHPV type 52. (**I**) Pseudovirus nAbs against HPV type 58. * *p* < 0.05, ** *p* < 0.01.

Cross-neutralizing antibody titers against HPVs (6, 11, 31, 33, 45, 52, 58) in rabbit immune sera collected on D61 ranged from 10^3^ to 10^6^ and were equivalent to those obtained for the Gardasil^®^9 group (*p* > 0.05) (Figure 3). In the Gardasil^®^9 group, however, the neutralizing antibody titers against HPV16 were 2.5 times higher than those in the LBTA group (GMT 80,767 vs. 32,366, *p* < 0.05), and those against HPV18 were 11.6 times higher (GMT 105,884 vs. 9165, *p* < 0.01) (Figure 3). Regardless, strong neutralizing antibody responses against HPV16 and HPV18 were observed in the LBTA group.

**Figure 4:**
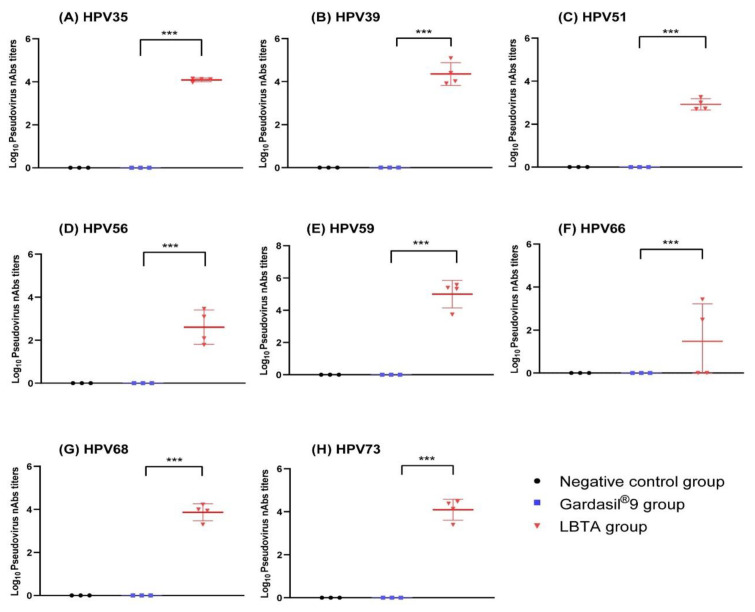
LBTA vaccination induced cross-neutralizing antibody responses in rabbits on D61 after primary vaccination against 8 HPV types not included in Gardasil^®^9. (**A**) Pseudovirus nAbs against HPV type 35. (**B**) Pseudovirus nAbs against HPV type 39. (**C**) Pseudovirus nAbs against HPV type 51. (**D**) Pseudovirus nAbs against HPV type 56. (**E**) Pseudovirus nAbs against HPV type 59. (**F**) Pseudovirus nAbs against HPV type 66. (**G**) Pseudovirus nAbs against HPV type 68. (**H**) Pseudovirus nAbs against HPV type 73. *** *p* < 0.001.

**Figure 5:**
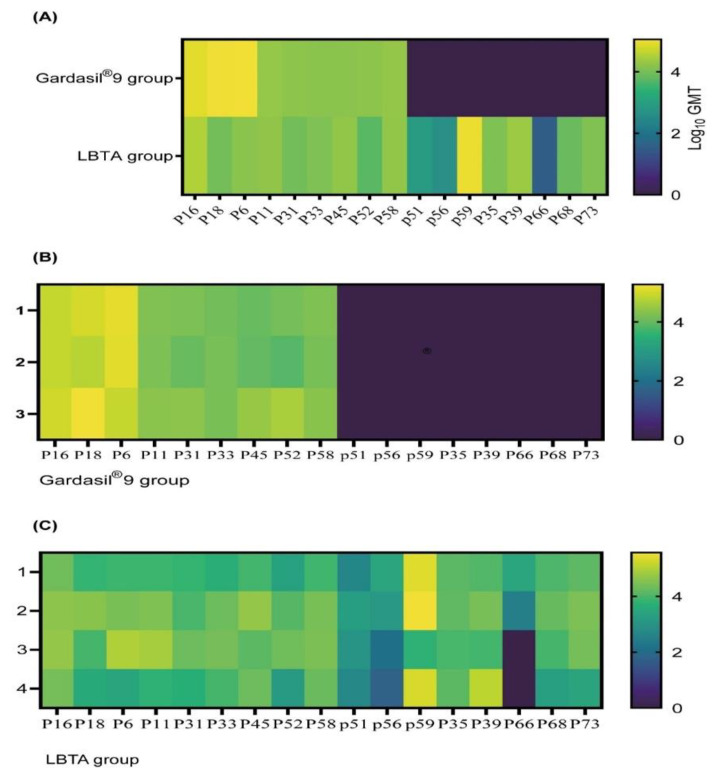
Heatmap representation of pseudovirus neutralizing titers of rabbits’ sera on D61 after primary immunization. (**A**) Heatmap representation of pseudovirus neutralizing GMTs in rabbits’ sera from the Gardasil^®^9 and LBTA groups. (**B**) Heatmap representation of pseudovirus neutralizing titers of rabbits’ sera from Gardasil^®^9 group. (**C**) Heatmap representation of pseudovirus neutralizing GMTs of rabbits’ sera from the LBTA group. Neutralizing antibody titers ranging from 50 to 187064 are color-coded from green to yellow; the black color indicates pseudovirus neutralizing titers < 50 (no neutralization). P stands for HPV.

Cross-neutralizing antibody responses against the other eight HPV pseudoviruses not covered by Gardasil^®^9 were also measured. As expected, Gardasil^®^9 did not induce neutralizing responses against HPVs (35, 39, 51, 56, 59, 66, 68, 73) (Figure 4). In contrast, LBTA vaccination induced significant neutralizing antibody titers against HPV35 (GMT, 12229), HPV39 (GMT, 22685), HPV51 (GMT, 833), HPV56 (GMT, 402), HPV59 (GMT, 99690), HPV66 (GMT, 30), HPV68 (GMT, 7304), and HPV73 (GMT, 12377). More specifically, GMTs against HPVs (35, 39, 59, 73) were higher than 10^4^. The heat map (Figure 5) shows rabbit-neutralizing antibody titers against 17 different HPV pseudoviruses, thus illustrating the broad spectrum of immune responses induced by LBTA immunization.

### 3.5. Validation of the Cervicovaginal Mouse Model for HPV Pseudovirus Challenge

We first established the mouse model for HPV16 pseudovirus vaginal infection (Supplementary Figure S1A). Vaginal cytology was used to determine the stage of the mice’s estrus cycle. Four days after the mice were injected with medroxyprogesterone acetate, vaginal smears (Supplementary Figure S1C) and HE staining (Supplementary Figure S1E) showed a high number of leukocytes, indicating that the mice were in diestrus. Results from three *in vivo* infection experiments showed that the average radiance values of the control group were 2.35 × 10^5^, 2.02 × 10^6^, and 9.02 × 10^5^ p/s/cm^2^/sr, respectively, which were reproducibly more than 100 times higher than those of the negative group (Supplementary Figure S1F). These results indicated that the model was valid and reliable and could be used for HPV pseudovirus challenge experiments against all 17 HPV types tested.

### 3.6. Passive Immunization with LBTA Antiserum Protected Mice against Cervicovaginal Challenge with 17 HPV Pseudovirions

Rabbits vaccinated with a three-dose regimen of adjuvated LBTA developed robust cross-protective immunity. We selected the D61 sera from rabbits vaccinated with either 125 μg LBTA and 500 μg AlPO_4_ or Gardasil^®^9 to compare their protective ability in passively immunized mice against vaginal challenge with 17 HPV pseudovirions. The sera from the Saline group were used as control.

Twenty-four female BALB/c mice were given medroxyprogesterone acetate before being divided into four groups of six mice. As illustrated in Supplementary Figure S2, all animals received a subcutaneous injection of 3 mg of medroxyprogesterone acetate on Day 0. The animals in the control group were not passively immunized with rabbit sera on Day 3, while the animals in the Saline group were passively immunized by intraperitoneal injection of 100 μL of sera from rabbits vaccinated with saline on Day 3. The animals in LBTA and Gardasil^®^9 groups were passively immunized by intraperitoneal injection of 100 μL of sera from rabbits vaccinated with LBTA or Gardasil^®^9 on Day 3, respectively. On Day 4, the mice were inoculated with either PsV 6, 11, 16, 18, 31, 33, 45, 52, 58, 51, 56, 59, 35, 39, 66, 68, or 73, encapsulating luciferase. On Day 6, vaginal infection was monitored using IVIS imaging of luciferase-based bioluminescence.

**Figure 6:**
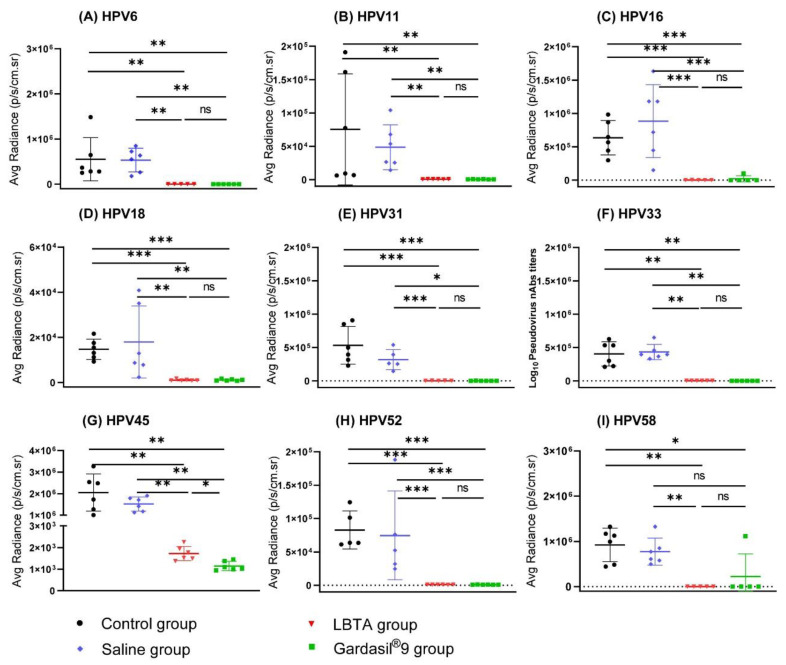
Vaccination with LBTA protected mice against challenges with the 9 HPV types included in Gardasil^®^9. (**A**) Average radiances in mice from control, Saline, LBTA, and Gardasil^®^9 groups challenged with HPV6 pseudovirion. (**B**) Average radiances in mice from control, Saline, LBTA, and Gardasil^®^9 groups challenged with HPV11 pseudovirions. (**C**) Average radiances in mice from control, Saline, LBTA, and Gardasil^®^9 groups challenged with HPV16 pseudovirions. (**D**) Average radiances in mice from control, Saline, LBTA, and Gardasil^®^9 groups challenged with HPV18 pseudovirions. (**E**) Average radiances in mice from control, Saline, LBTA, and Gardasil^®^9 groups challenged with HPV31 pseudovirions. (**F**) Average radiances in mice from control, Saline, LBTA, and Gardasil^®^9 groups challenged with HPV33 pseudovirions. (**G**) Average radiances in mice from control, Saline, LBTA, and Gardasil^®^9 groups challenged with HPV45 pseudovirions. (**H**) Average radiances in mice from control, Saline, LBTA, and Gardasil^®^9 groups challenged with HPV52 pseudovirions. (**I**) Average radiances in mice from control, Saline, LBTA, and Gardasil^®^9 groups challenged with HPV58 pseudovirions. * *p* < 0.05, ** *p* < 0.01, *** *p* < 0.001. ns—not significant.

The passive transfer of rabbit antisera from both the LBTA and Gardasil^®^9 groups resulted in a significant reduction in bioluminescence signals after cervicovaginal challenge with the nine HPV types included in Gardasil^®^9 compared to the control group or the Saline group (Figure 6). There was no significant difference between the LBTA and Gardasil^®^9 groups in providing protection against HPVs (6, 11, 16, 18, 31, 33, 52, 58). However, for the HPV45 challenge, mice immunized with LBTA showed a slightly higher average radiance compared to those immunized with Gardasil^®^9 (*p* < 0.05) (Figure 6G). Overall, both LBTA and Gardasil^®^9 provided comparable and significant protection against the nine Gardasil^®^9 HPV types.

**Figure 7:**
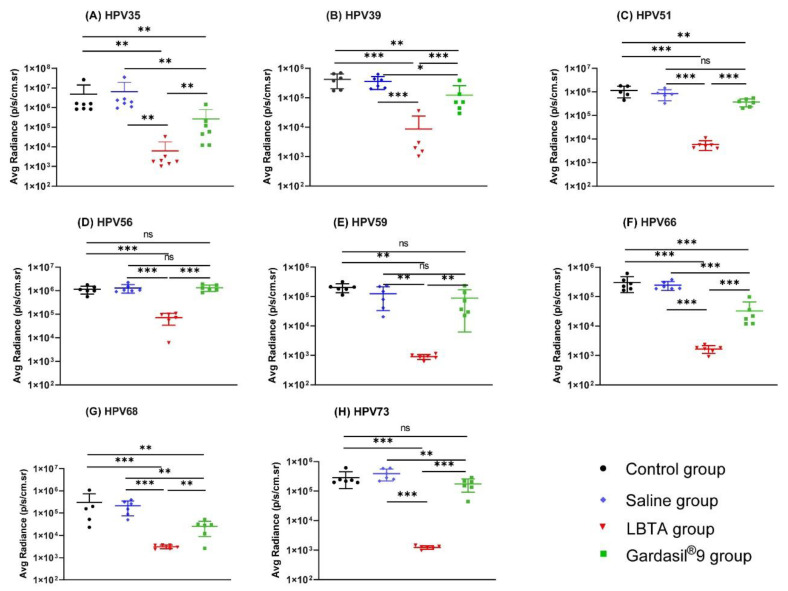
Vaccination with LBTA protected mice against challenge with 8 HPV types not included in Gardasil^®^9. (**A**) Average radiances in mice from control, Saline, LBTA, and Gardasil^®^9 groups challenged with HPV35 pseudovirions. (**B**) Average radiances in mice from control, Saline, LBTA, and Gardasil^®^9 groups challenged with HPV39 pseudovirions. (**C**) Average radiances in mice from control, Saline, LBTA, and Gardasil^®^9 groups challenged with HPV51 pseudovirions. (**D**) Average radiances in mice from control, Saline, LBTA, and Gardasil^®^9 groups challenged with HPV56 pseudovirions. (**E**) Average radiances in mice from control, Saline, LBTA, and Gardasil^®^9 groups challenged with HPV59 pseudovirions. (**F**) Average radiances in mice from control, Saline, LBTA, and Gardasil^®^9 groups challenged with HPV66 pseudovirions. (**G**) Average radiances in mice from control, Saline, LBTA, and Gardasil^®^9 groups challenged with HPV68 pseudovirions. (**H**) Average radiances in mice from control, Saline, LBTA, and Gardasil^®^9 groups challenged with HPV73 pseudovirions. * *p* < 0.05, ** *p* < 0.01, *** *p* < 0.001. ns—not significant.

Compared with the control and Saline groups, the passive transfer of antisera from the LBTA group significantly reduced the bioluminescence signals following cervicovaginal challenge with HPV35, HPV39, HPV51, HPV56, HPV59, HPV66, HPV68, and HPV73 serotypes that are not included in Gardasil^®^9 (Figure 7).

The bioluminescence signals in the Gardasil^®^9 group were also significantly reduced after the challenge with HPV35, HPV39, HPV66, and HPV68 but not after the challenge with HPV56 and HPV59 (*p* > 0.05). The average radiance in the Gardasil^®^9 group was significantly reduced (400,040) after the challenge with HPV51 compared with the control group (1,014,650, *p* < 0.01) but not when compared with the Saline group (841,600, *p* > 0.05) (Figure 7C). In contrast, the average radiance of the Gardasil^®^9 group after the HPV73 challenge (174,202) was comparable to that of the control group (285,783, *p* > 0.05) but was moderately reduced compared with the Saline group (392,017, *p* < 0.01) (Figure 7H). However, in both instances, radiance values are of the same order of magnitude, and statistical results might be biased due to the limited number of animals studied. Globally, however, results from passive immunization experiments with sera from animals vaccinated with the nonavalent vaccine showed that Gardasil^®^9 was able to elicit a certain level of cross-protection against HPV35, HPV39, HPV66, and HPV68 serotypes that are not included in the vaccine.

Passive immunization with sera from LBTA-vaccinated rabbits substantially reduced the average radiance signals in mice challenged with the eight HPV types not included in the commercial vaccine markedly more than sera from the Gardasil^®^9-vaccinated group (HPV35, *p* < 0.01; HPV39, *p* < 0.001; HPV51, *p <* 0.001; HPV56, *p* < 0.001; HPV59, *p* < 0.01; HPV66, *p* < 0.001; HPV68, *p* < 0.01; HPV73, *p* < 0.001) (Figure 7A–H). These results clearly demonstrate that passive immunization with antisera from rabbits immunized with LBTA was significantly more effective than Gardasil^®^9 at protecting mice against infection with the eight HPV types not included in the nonavalent vaccine.

## 4. Discussion

The licensed bivalent, quadrivalent, and nonavalent HPV vaccines are safe and highly efficacious [18,40]. However, in spite of some cross-reactivity, L1-based vaccines primarily provide type-specific protection against a limited number of HPV types, including HPV16 and HPV18, which are responsible for 70% of all cervical cancers. However, several other HPV types not included in licensed vaccines can also lead to cervical cancer and other benign and malignant HPV-related diseases. Studies on the incremental costs and benefits of additional serotypes in the nonavalent vaccine over the quadrivalent vaccines showed that immunization of both sexes with Gardasil^®^9 in the USA could improve health outcomes while being cost-saving [41]. However, the production of multivalent L1 VLP-based vaccines to broaden HPV type coverage is complex and costly, regardless of the manufacturing process. The cost-effectiveness of multivalent VLP-based vaccines remains uncertain for certain low- and middle-income countries [24,42]. A study comparing the nonavalent and bivalent HPV L1 VLP vaccines in 48 Gavi-eligible countries found that while the nonavalent vaccine is projected to prevent more cervical cancer cases, the bivalent vaccine with strong cross-protection could offer considerable health benefits and be a more cost-effective option for many of these countries [43]. For example, Gardasil^®^9 is not cost-effective under any modeling scenario in the Philippines [25]. Thus, cost remains a major barrier to vaccine implementation in LMICS, and there is a need for a new generation of simpler, cheaper, and broader-spectrum vaccines against mucosal and cutaneous HPVs [44].

Although current L1 VLP-based vaccines confer some degree of cross-protection against highly phylogenetically related non-vaccine HPV types [45,46], they primarily protect against the vaccine genotypes. The highly conserved L2 capsid protein has thus been proposed as a suitable alternative immunogen to produce pan-HPV prophylactic vaccines against a larger spectrum of high- and low-risk αHPVs and βHPVs not covered by Gardasil^®^9 [47]. Campo et al. [48] were the first to show that N-terminal L2 peptides fused to glutathione-S transferase (GST) conferred a high degree of protection against the bovine mucosal virus BPV-4. Subsequently, it was reported that immunization with the N-terminal region of HPV L2 that contains linear cross-neutralization epitopes elicited broadly neutralizing responses against mucosal and cutaneous papillomaviruses and conferred cross-protection against highly divergent HPVs [48,49]. Furthermore, vaccination of healthy volunteers with an HPV16 L2E7E6 fusion protein was shown to elicit antibody responses that cross-neutralized several papillomavirus species. Current L2-based vaccine strategies under investigation include the development of hybrid L1/L2 VLPs containing either the full-length L2 or L2 neutralization epitopes inserted in the L1 capsid, L2 cross-protective epitopes displayed on other eukaryotic virus capsids or bacteriophage coat proteins, L2 epitopes fused to thioredoxin or immunostimulatory ligands, and concatemers of cross-neutralization epitopes [26,28].

The L2 N-terminal region encompassing residues 11 to 88 becomes exposed after the virion binds to the epithelial basal membrane. It contains several linear, overlapping, and highly conserved cross-neutralization B-cell epitopes among divergent HPV isolates (aa17–36, 20–38, 32–51, 49–71, 56–75, 58–64, 63–89, 64–73, 65–81, 69–81) [30,35,48,50,51,52,53] that are recognized by anti-L2 polyclonal and/or monoclonal neutralizing antibodies [35,53]. Regardless of the vaccine platform used, immunization with a single conserved L2 neutralization epitope, in particular the RG1 epitope (aa17–36) [54,55], is able to induce robust neutralizing responses in mice and rabbits and cross-protection against multiple divergent mucosal and cutaneous HPV isolates [28,56]. The breadth and magnitude of cross-protective immune responses are increased when more than one neutralization epitope or a mixture of the same epitope from different serotypes or multimers of single epitopes are used as immunogens [30,57,58,59]. Immunization with these epitope-based vaccines not only induced protection against multiple sexually transmitted oncogenic and benign αHPV types but also against non-sexually transmitted HPVs associated with cutaneous warts and non-melanoma skin cancer [55,60,61].

To achieve broad HPV protection with a cost-effective single-chain vaccine, Jagu et al. [32] engineered and evaluated two L2 multimeric proteins, a11–88x5 and a11–88x8, comprising L2 residues 11–88 from five HPV (6, 16, 18, 31, 39) and eight HPV (6, 16, 18, 31, 39, 51, 56, 73) divergent isolates, respectively. The LTBA drug product has very similar properties to the a11–88x5 antigen, although it was generated with a distinct production procedure compared to the earlier product, which may influence its stability and immunogenicity. The a11–88x5 vaccine adjuvanted in alum induced similar neutralizing antibody responses against eleven HPVs from six different alphapapillomavirus species (α 5, 6, 7, 9, 10, 11) and the same level of protection against murine vaginal challenge with low-risk HPV6 and ten oncogenic HPV (16, 26, 31, 33, 35, 45, 51, 56, 58, 59) serotypes. The a11–88x5 multimer, alone or fused to flagellin, also protected rabbits against cutaneous challenge with HPV (6, 16, 18, 31, 45, 58) quasi-virions [58]. Furthermore, unlike L1-based vaccines, the L2 a11–88x5 concatemer, which contains neutralization epitopes conserved across papillomavirus species, was shown to neutralize several βHPVs and protect against all βHPV tested [62], including βHPV5, which is the dominant type associated with cutaneous squamous-cell carcinoma in epidermodysplasia verruciformis and immunocompromised patients [26].

The objective of this study was to produce the recombinant L2 a11–88x5 multimeric vaccine (LBTA DP) in *E*. *coli* under cGMP conditions, characterize it, assess its stability and safety, and evaluate the magnitude and breadth of its cross-protective ability compared to those of a full human dose of Gardasil^®^9. The 40 kDa protein was purified to homogeneity by chromatography with a purity ≥ 90% as judged by SDS PAGE and reverse RP-HPLC chromatography. In spite of the presence of 10 cysteines in the construct, analysis by SDS-PAGE under non-reducing conditions revealed the absence of covalent multimerization that could have arisen from potential inter-chain disulfide bond formation. We did not investigate how many of the cysteines at positions 22 and 28 were free or involved in intra-chain disulfide bond formation. Although reduction of the Cys22-Cys28 loop does not affect epitope (aa17–36) recognition by RG1 monoclonal antibody, it may impact its immunogenicity [57]. The JWW1 cross-neutralizing monoclonal antibody to L2 epitope aa17–36 from HPV 16, 18, 31, and 39 and JWW2 cross-neutralizing monoclonal antibody to L2 aa58–64 from HPV 6, 16, 18, 31, and 39 recognized the LBTA [35], providing an *in vitro* surrogate to assess its antigenicity.

Helper T-cell responses are important to promote robust and durable serum IgG antibody levels. We were thus curious as to whether the L2 N-terminal sequence (aa11–88) from vaccine types contains MHC Class II-restricted T-cell epitopes [63,64]. In silico analysis using the IEDB NetMHCIIpan 4.1 prediction software suggests that LBTA harbors helper T-cell epitopes restricted by the supertype alleles HLA-DRB1 11:01, HLA-DRB1 15:01, HLA-DRB4 01:01, HLA-DRB5 01:01, and HLA-DQA1 05:01/DQB103:01.

An L2-based preventive vaccine has not been developed commercially, most likely because it is considered a weaker immunogen than L1 and lacks VLP structure on its own [65]. Vaccination with L2 has resulted in lower neutralizing titers than with L1 [66], but these low titers are sufficient for protection [66]. Previous active vaccination studies had shown that the a11–88x5 (HPV6, 16, 18, 31, 39) immunogen elicited robust protective responses in mice against intravaginal challenge with 11 medically significant αHPV species (HPV6, 16, 26, 31, 33, 35, 45, 51, 56, 58, 59). However, the HPV16 neutralizing titers induced in rabbits by the a11–88x5 immunogen adjuvanted in alum were two orders of magnitude lower than those elicited by bivalent Cervarix^®^ or quadrivalent Gardasil^®^ [32]. It is important to note that these studies utilized the first-generation neutralization assay [67] that is poorly suited to measure L2-specific neutralizing antibodies because it poorly represents some aspects of the early events of infection, notably extracellular furin processing of virions on the basement membrane [29,68].

Since these early studies, a new format of *in vitro* neutralization assays was developed on furin pre-cleaved pseudovirus [69,70,71]. We assessed the cross-type protective ability of clinical-grade LBTA and compared it with that of Gardasil^®^9 using the improved neutralization assay based on furin pre-cleaved virus [71]. We found that immunization of rabbits with 125 µg of LBTA adsorbed onto 500 µg of alum elicited strong cross-neutralizing responses against the nine HPV types (6, 11, 16, 18, 31, 33, 45, 52, 58) present in Gardasil^®^9 equivalent to those induced by a full human dose of the licensed vaccine except for anti-HPV18 neutralizing antibody titers, which were still high but one order of magnitude lower. Interestingly, anti-HPV16 neutralization titers were almost two orders of magnitude higher than those previously reported by Jagu et al. [32] for their a11–88x5 construct because of the use of the improved assay format [71]. Moreover, LBTA antiserum strongly neutralized eight additional HPV types (35, 39, 51, 56, 59, 66, 68, 73), whereas Gardasil^®^9 did not.

In passive immunization experiments that are more sensitive than the HPV pseudovirion neutralization assay, Gardasil^®^9 antiserum was able to confer some degree of cross-protection against some non-vaccine HPV strains [34,38,66,68], although to a significantly lower level than LBTA. Passive transfer of Gardasil^®^9 antiserum did not protect against HPV 51, 56, 59, or 73 challenge. In contrast, a11–88x5 antisera was protective against vaginal challenge with the 11 HPV types (6, 16, 26, 31, 33, 35, 45, 51, 56, 58, 59) tested [32]. Here, we showed that passive transfer of LBTA immune sera completely protected naïve mice against a broad spectrum of 17 αHPV types, including six additional types of HPV (18, 39, 52, 58, 66, 68, 73), although HPV26 was not tested here. Combining our data with those of Jagu et al. [32] and Olczak et al. [62], we can reasonably expect that the LBTA vaccine will protect mice against vaginal challenge with 18 αHPV types and cutaneous challenge with 4 βHPV types (Supplementary Table S5). Additional studies are ongoing to assess the full extent of the cross-protective ability of LTBA against mucosal and cutaneous HPVs.

Besides its potential advantage over licensed L1 VLP vaccines by broadening HPV coverage, LBTA is a single-chain concatemer of linear neutralization epitopes produced in a simple high-yield *E. coli* expression system and may thus be produced at a significantly lower cost [66,72] than the polyvalent VLP-based vaccines made in yeast or baculovirus. Furthermore, the glycosylation patterns of VLPs differ between humans and yeast, which may have implications in terms of protein immunogenicity, solubility, and stability [73], whereas the expression of Cervarix^®^ in insect cells is associated with low protein expression levels and high production costs [74].

In conclusion, we have produced three clinical lots of a single-chain multimeric HPV vaccine in a simple *E. coli* expression system, adjuvanted in aluminum phosphate, which is well accepted for human use. The lyophilized cGMP vaccine is thermostable and has a good safety profile in preclinical models. It has not only been shown to be as potent and protective as Gardasil^®^9 in animal challenge models, but LBTA also to confer significantly broader cross-protection efficacy than the nonavalent vaccine against 17 αHPVs and potentially against 18 αHPV and 4 βHPV types. These findings highlight LBTA’s promises as a next-generation HPV vaccine with comprehensive coverage to reduce economic and health care burden for cervical cancer prevention in LMICs in particular and to provide a promising avenue for further vaccine developments and clinical applications. It has received ethical approval from the Human Research Ethics Committee (HREC) and been acknowledged by the Therapeutic Goods Administration (TGA) of Australia and is thus officially approved for first-in-human clinical trials (NCT05672966).

## Data Availability

The raw data supporting the conclusions of this article will be made available by the authors without undue reservation.

## Supplementary Materials

The following supporting information can be downloaded at https://www.mdpi.com/article/10.3390/vaccines12060689/s1, Table S1: Dose of HPV pseudovirus used in the mouse cervicovaginal challenge model. Table S2: Stability of LBTA cGMP drug substance stored at ≤−60 °C. Table S3: Stability of LBTA cGMP drug product (lyophilized powder) stored at 5 ± 3 °C. Table S4: Summary of LBTA drug product (240 µg per dose) in-use stability results. Table S5: HPV types tested against which Gardasil^®^9, a11–88x5 multimer, or LBTA vaccines conferred protection in the mouse cervicovaginal challenge model. Supplementary Figure S1: Establishment of the mouse model for HPV pseudovirus vaginal challenge. (A) Schedule of the *in vivo* HPV infection model in mice. (B) Vaginal smear of mice from negative group. (C) Vaginal smear of mice from control group. (D) HE staining of mouse vaginal smear from negative group. (E) HE staining of mouse vaginal smear from control group. (F) Average radiance data from three trials. Supplementary Figure S2: Schedule of passive immunization in the mouse *in vivo* HPV vaginal infection model. (A) Control group’s schedule of passive immunization in the mouse *in vivo* HPV vaginal infection model. (B) Saline group’s schedule of passive immunization in the mouse *in vivo* HPV vaginal infection model. (C) LBTA group’s schedule of passive immunization in the mouse *in vivo* HPV vaginal infection model. (D) Gardasil^®^9 group’s schedule of passive immunization in the mouse *in vivo* HPV vaginal infection model. Supplementary Figure S3: Transmission electron micrograph of HPV16 pseudovirions (PsV) produced in HEK293TT cells. The PsV size is about 60 nm. References [32,62] are cited in the Supplementary Materials.
