# Supplementary material for: Vaccination with a Human Papillomavirus L2 Multimer Provides Broad Protection against 17 Human Papillomavirus Types in the Mouse Cervicovaginal Challenge Model
Source: Vaccines (Basel). 2024 Jun 20;12(6):689. doi: 10.3390/vaccines12060689 (PMC11209485; doi:10.3390/vaccines12060689)
Supplement: Supplementary file 1 [file vaccines-12-00689-s001.zip › vaccines-2999501-supplementary.pdf]

Supplementary Tables

Table S1. Dose of HPV pseudovirus used in the mouse cervicovaginal challenge model.

HPV type	Dose/animal ($TCID_{50}$)
HPV16	1.50×10^7
HPV18	8.43×10^6
HPV6	1.50×10^7
HPV11	8.43×10^6
HPV31	6.32×10^6
HPV59	6.32×10^6
HPV39	2.67×10^7
HPV51	4.74×10^7
HPV56	2.67×10^7
HPV35	2.00×10^7
HPV33	8.43×10^6
HPV45	1.50×10^7
HPV52	6.32×10^6
HPV58	6.32×10^6
HPV66	3.56×10^7
HPV68	6.32×10^7
HPV73	6.32×10^7

Table S2. Stability of LBTA cGMP drug substance stored at ≤−60°C.

Lot #	Time of storage (at ≤-60°C)	Results		
		Protein content (≥500μg/mL*)	Purity SDS PAGE (≥85%*)	Purity HPLC (≥90%*)
HPV-P20220711	Time zero	1247	98%	100%
	3 months	1213	100%	100%
	6 months	1153	94%	86%
HPV-P20220812	Time zero	1082	92%	100%
	3 months	1060	100%	100%
	6 months	1244	98%	100%
HPV-P20220813	Time zero	1239	90%	100%
	3 months	1213	100%	98%
	6 months	1257	94%	100%

*Acceptance criterion.

Table S3. Stability of LBTA cGMP drug product (lyophilized powder) stored at 5 ± 3 ℃.

Lot #	Time of storage (at 5°C±3°C)	Results			
		Protein content (240 µg +/- 60 µg/dose*)	Adsorption (≥80%*)	Purity by SDS PAGE (≥85%*)	Purity by RP-HPLC (≥90%*)
HPV20220803 0.5ml/vial	Time zero	203	96%	100%	100%
	1 month	203	97%	100%	100%
	3 months	217	96%	100%	100%
	6 months	222	N/A	98%	100%
HPV20220904 0.5ml/vial	Time zero	196	97%	100%	100%
	1 month	216	98%	100%	100%
	3 months	207	97%	97%	100%
	6 months	225	N/A	97%	100%
HPV20220905 0.5ml/vial	Time zero	227	98%	100%	100%
	1 month	227	97%	100%	100%
	3 months	245	98%	100%	100%
	6 months	242	N/A	93%	100%

* Acceptance criterion

N/A: not available

Table S4. Summary of LBTA drug product (240 µg per dose) in-use stability results.

HPV cGMP lots	In-use condition	Time (Hour)	Protein Content (µg/dose)	$AlPO_4$ Adsorption (%)
HPV Lot #1		0.0	268	97
	5°C±3°C	0.5	266	97
		1.0	274	97
		2.0	266	98
		4.0	258	98
	25°C±2°C	0.5	265	97
		1.0	273	97
		2.0	274	98
	37°C±2°C	0.5	270	97
		1.0	271	97
		2.0	268	98
HPV Lot #2		0	258	96
	5°C±3°C	0.5	267	97
		1.0	266	97
		2.0	259	98
		4.0	256	98
	25°C±2°C	0.5	269	98
		1.0	274	97
		2.0	265	98
	37°C±2°C	0.5	269	98
		1.0	274	97
		2.0	265	98
HPV Lot #3		0	294	97
	5°C±3°C	0.5	296	97
		1.0	293	97
		2.0	303	98
		4.0	274	98
	25°C±2°C	0.5	286	98
		1.0	294	98
		2.0	306	98

HPV cGMP lots	In-use condition	Time (Hour)	Protein Content (µg/dose)	$AlPO_4$ Adsorption (%)
	37°C±2°C	0.5	290	98
		1.0	293	97
		2.0	306	98

Table S5. HPV serotypes tested against which Gardasil®9, a11-88x5 multimer or LBTA vaccines conferred protection in the mouse cervicovaginal challenge model.

	Alpha HPVs																		Beta HPVs			
Gardasil®9	6	11	16	18		31	33															
α11-88x5*	6		16		26	31	33	35		45	51		56		59							
α11-88x5**																			5	49	76	96
LBTA	6	11	16	18		31	33	35	39	45	51	52	56	58	59	66	68	73				

* Jagu *et al.* [32]

**Olczak *et al.* [62]

Supplement Figures:

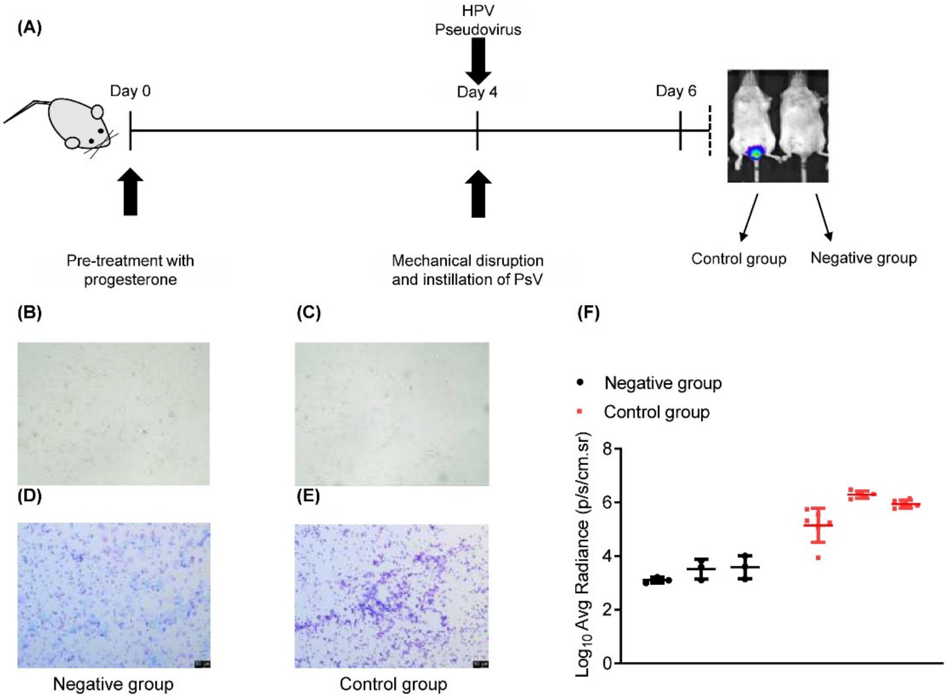

Supplementary Figure S1. Establishment of the mouse model for HPV pseudovirus vaginal challenge. **(A)** Schedule of the *in vivo* HPV infection model in mice. **(B)** Vaginal smear of mice from negative group. **(C)** Vaginal smear of mice from control group. **(D)** HE staining of mouse vaginal smear from negative group. **(E)** HE staining of mouse vaginal smear from control group. **(F)** Average radiance data from three trials.

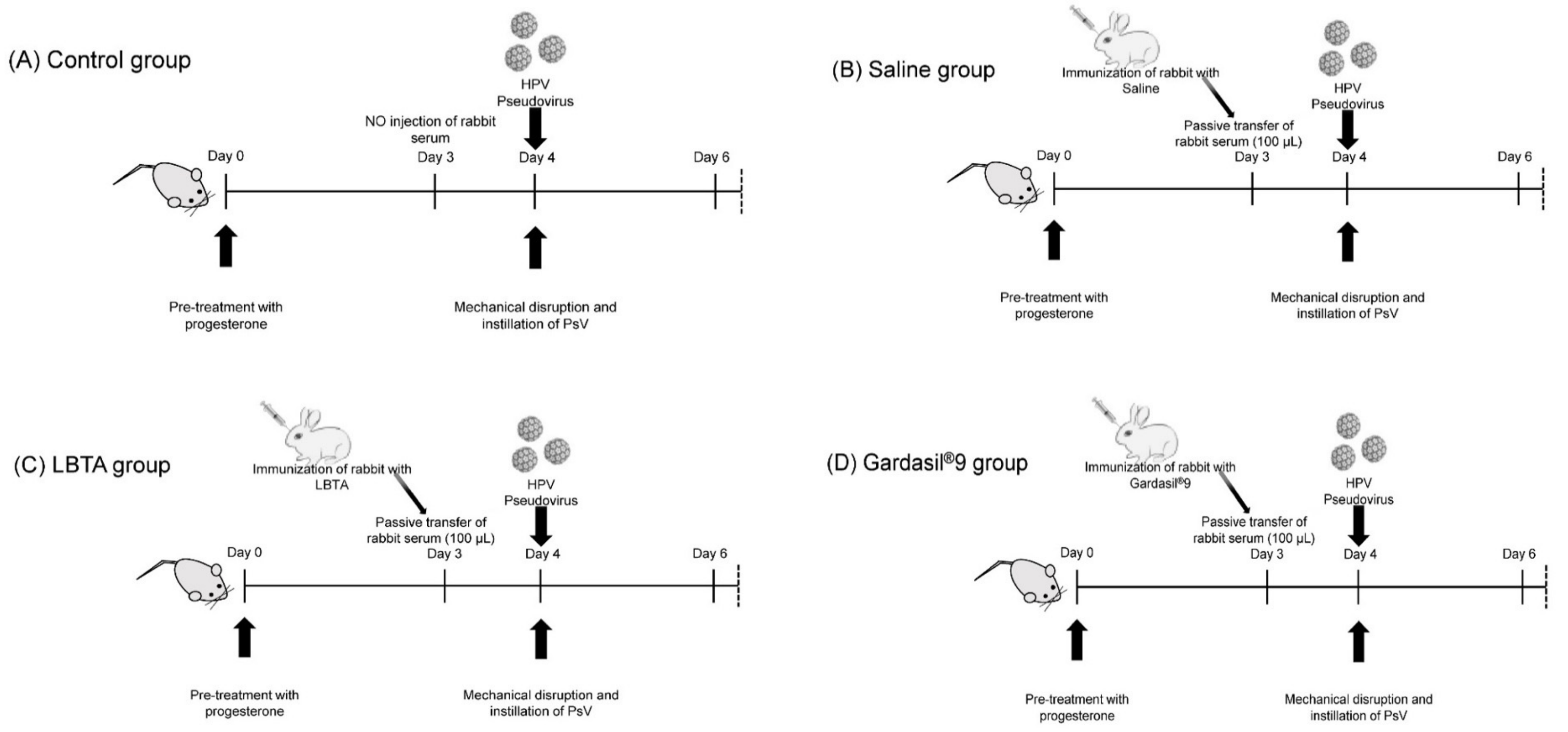

Supplementary Figure S2. Schedule of passive immunization in the mouse *in vivo* HPV vaginal infection model. **(A)** Control group's schedule of passive immunization in the mouse *in vivo* HPV vaginal infection model. **(B)** Saline group's schedule of passive immunization in the mouse *in vivo* HPV vaginal infection model. **(C)** LBTA group's schedule of passive immunization in the mouse *in vivo* HPV vaginal infection model. **(D)** Gardasil®9 group's schedule of passive immunization in the mouse *in vivo* HPV vaginal infection model.

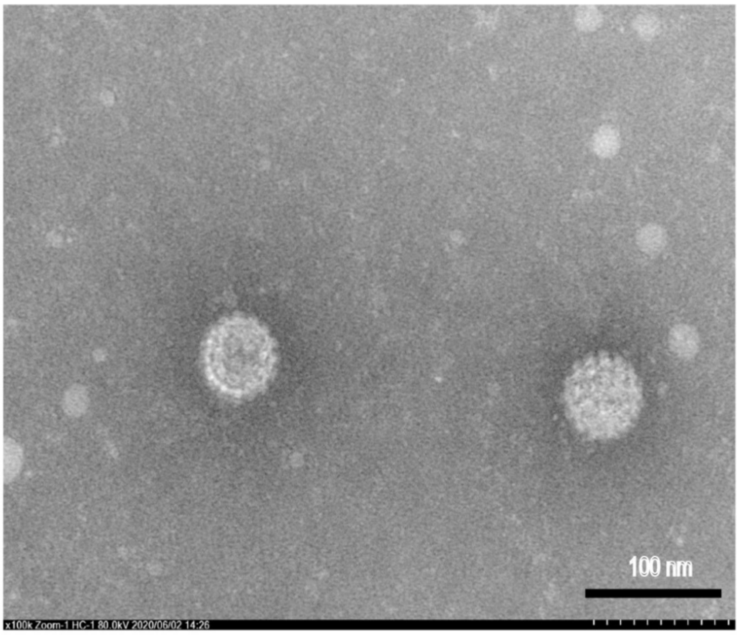

Supplementary Figure S3. Transmission electron micrograph of HPV16 pseudovirions (PsV) produced in HEK293TT cells. The PsV size is about 60 nm.
